# Continuing Education for Haitian Nurses: Evidence from Qualitative and Quantitative Inquiry

**DOI:** 10.5334/aogh.2538

**Published:** 2019-07-01

**Authors:** Jill Caporiccio, Kettie R. Louis, Annie Lewis-O’Connor, Kerry Quealy Son, Nadia Raymond, Isis A. Garcia-Rodriguez, Emily Dollar, Laura Gonzalez

**Affiliations:** 1 Massachusetts General Hospital, US; 2 Boston Medical Center, US; 3 Brigham and Women’s Hospital, US; 4 Boston Healthcare for the Homeless, US; 5 UMass Lowell, US; 6 Suffolk University, US; 7 Geisel School of Medicine at Dartmouth, US; 8 Boston College, US

## Abstract

**Background:**

There is increasing evidence that to improve nursing practice, nurses must embrace lifelong learning. Research indicates that engagement in lifelong learning positively affects the quality of nursing care, improves patient outcomes, and increases nurses’ job satisfaction. Both lack of standardized initial education and nurses’ limited opportunities for lifelong learning are challenges in Haiti. It is crucial to ensure adequate continuing education in order to support the professional growth and development of Haiti’s existing nursing workforce.

**Objectives:**

The objectives of this study were to: 1) assess the continuing education nursing needs and desires of practicing Haitian nurses and 2) contribute to the body of knowledge about nursing in Haiti to help inform practice and policy.

**Methods:**

A multimodal needs assessment approach was used, with semi-structured focus groups and written surveys. The results were analyzed, and common themes were identified.

**Findings:**

The results were analyzed from 100 surveys and four focus groups (n = 33). Overwhelmingly, Haitian nurses desire continuing nursing education. Major themes include: recognition that continuing education is necessary to provide high quality patient care, continuing education saves lives, and more consistent and standardized initial nursing education is needed. Barriers to participation in continuing education opportunities were also identified.

**Conclusions:**

This study was one of the first formal studies that addressed continuing education needs of Haitian nurses. By identifying the barriers to important resources, we hope to continue to collaborate with our Haitian nursing colleagues to build curriculum and improve education programs. We also hope that this research will ensure that Haitian nurses voices are heard and will serve to foster change within the Haitian nursing education system. These results were shared with our nurse colleagues in Haiti.

## Introduction

Continuing education (CE) for healthcare providers is essential for their professional growth and development, as well as for the improved health outcomes of their patients. Strong healthcare systems require a variety of approaches to ensure that comprehensive health services are provided and that those services foster the delivery of evidenced-based health care. Nurses are key providers of healthcare delivery, comprising 60–80 percent of the total health care system’s workforce and providing 90 percent of all health care services worldwide [1].

Advancement in nursing education and the promotion of a well-educated workforce of nurses have been positively associated with improved health outcomes and decreased morbidity and mortality rates among patients [2,3,4,5,6,7]. Research has supported these outcomes and also suggest that scholars of nursing increasingly view lifelong learning as a pathway to competent and professional nursing [2,3,4,5,6,7,8]. In nursing, lifelong learning is achieved through continuous nursing education, which provides nurses with current knowledge and standards of care in their areas of practice. Continuing education should be a main focus for nurses everywhere to ensure stronger healthcare systems with better outcomes [9].

## Background

Haiti relies heavily on nurses to deliver healthcare. However, unlike countries such as the United States, in Haiti there is no formal infrastructure to ensure that currently practicing nurses have access to continuing education opportunities or professional advancement. Upon graduation from nursing programs, many nurses in Haiti do not have opportunities for continued learning. This research focused on Haitian nurses’ attitudes and knowledge relevant to continuing education in their home country. This research was supported by EqualHealth, a non-profit organization that ensures access to continuing education by Haitian healthcare professionals and institutions.

Haiti is the first independent black nation in the Western hemisphere whose independence was gained as part of a successful slave rebellion. Unfortunately, since its 1804 Revolution, poor trade policies, effects of colonialism, and political unrest have continued to affect Haiti and negatively impacts its development [10]. Haiti continues to experience many economic and social challenges including high unemployment rates, lack of access to clean water, poverty, and unstable housing [11] that affect its inhabitants and healthcare systems. In 2003, a Red Cross study found fewer than one hospital bed for every one thousand patients throughout Haiti and only 209 surgical beds for the six million people living outside of Port-au-Prince [12]. The devastating earthquake of 2010 only served to exacerbate conditions and challenges in Haiti. In October, 2016 Haiti was besieged by another natural disaster, Hurricane Matthew, which left more than 900 dead [13] and destroyed the health care infrastructures in southern Haiti. The challenges to providing health care services in the context of multiple setbacks caused by natural disasters are daunting.

Haiti does not have reliable statistics on its health workforce; but based on most current data, prior to the earthquake in 2010, there were only 5,400 health care providers living in Haiti [14]. Of this total, 1,400 of these providers were nurses and 1000 were nurse Auxiliaries (Nurse Auxiliaries are nurses who practice at a level similar to that of a licensed practical nurse in the United States) [15]. The nurse to inhabitant ratio in Haiti is 2.4 per 10,000 which is far less than other Caribbean nations, such as Jamaica, for example, where the nurse-to-inhabitant ratio is 15 per 10,000 [16] contrasting that to the United States, the ratio is 92.09 to 10,000 [17]. Adding to the lack of health care personnel, the 2010 earthquake destroyed many of Haiti’s schools, including the national nursing school in Port-au-Prince. According to the Ministry of Health (MOH) in Haiti in 2015, many new schools have been opened since that time and there now exist more than 400 private nursing schools, but it is not clear whether all offer high-quality nursing education [18].

Even with the increased number of nursing schools, it is believed that the number of healthcare personnel has only decreased since the earthquake. There is notably a shortage of healthcare providers, specifically nurses, throughout Haiti, even as the healthcare system relies heavily on nurses to deliver care. In many rural communities where there are no physicians, nurses are the only healthcare providers caring for most the population [19]. In addition to the shortage of nurses, there are challenges unique to Haitian nurses: poor working conditions, lack of professional autonomy, inadequate pay, and a health care system challenged because of pervasive poverty and lack of resources. Additionally, nurses are often working in environments with little interdisciplinary collaboration and opportunities for advancement and have little to no training following their initial nursing school education. Many of the educational resources available in Haiti are outdated and not applicable to low-resource settings. Since conducting this research, we have found some institutions in Haiti that have initiated educational programs and are providing continuing nursing education to their staff; we found these institutions, however to be the exceptions.

## History of Continuing Education

Continuing education for nurses in the United States dates back to the 1800’s and the work of Florence Nightingale. Nightingale made a statement that captures the philosophy of continuing nursing education, “Let us never consider ourselves as finished nurses…we must be learning all our lives”. Health providers deliver care based on scientific research, which is ever evolving. CE is a standard of practice in US nursing and is often a condition for re-licensure (states laws may vary from state to state). In short, continuing education is a way to assure that nursing is informed of current evidence that informs clinical practice.

Continuing education should last the duration of a nurse’s career and serve two functions: the maintenance of current practice and the translation of knowledge into practice. Research shows that engagement in lifelong learning affects the quality of nursing care and improves patient outcomes, as well as increases job satisfaction and improves nurses’ perception of self-efficacy [20,21]. However, in low resource settings, the use of continuing nursing education to strengthen the healthcare delivery has not been utilized to its capacity [19]. This research highlights the interest, challenges and barriers to continuing education for Haitian nurses.

Nursing education in Haiti has been historically challenging and inadequate for nurses. Many nursing institutions lack basic teaching tools to educate their students such as: textbooks, simulation laboratories, computer access, and libraries. Nursing educators in Haiti have been given a minimum of formal education, often possessing only an Associate Degree or Diploma after completing two years of studies and one year of social service like a residency program. Lack of qualified nursing faculty has resulted in nursing students being taught by medical doctors [22]. Until recently very few educators had advanced degrees [23] unless they were educated overseas. Currently two universities are graduating nurses with baccalaureate degrees and in collaboration with foreign institutions some nurses in Haiti are now getting their Masters.

Nursing education has also been affected by structural and economic factors that have a direct impact on nursing practice and health care outcomes. These include limited access to technology, limited funds for current teaching materials, lack of qualified faculty, inadequate clinical sites and clinical instructors, and overcrowded classrooms [3,9,20]. In addition, there is a lack of regulation for non-credentialed nursing schools whose graduates are not prepared for practice [2,5]. In 2014, the United States Agency International Development (USAID) funded Health Financing and Governance (HFG) project confirmed that there are four hundred (400) private nursing schools in operations throughout Haiti [24]. The growth in number of private nursing schools places the public at risk as not much is known about the training that the students receive at these institutions. The HFG project worked with the Ministry of Health (MOH) to strengthen its ability to manage nursing educational institutions through a system known as “reconnaissance”. Reconnaissance corresponds to an accreditation where a neutral party evaluates strengths, weaknesses and possibilities for improvement within an educational system. In Haiti, the reconnaissance project goal was to improve the quality of nursing education, protect the public, guarantee patient safety and ensure that program teaches appropriate competencies for entry to practice [24].

## Methods

Approval to conduct this research was obtained from the institutional review boards (IRB) of Partners Healthcare, Boston Medical Center, Simmons College and the Ministry of Health in Haiti. EqualHealth’s nursing research team traveled to four different hospitals (public, private, and non-governmental organizations (NGO) hospitals) throughout the country. This study utilized a mixed method design including staff surveys and focus groups in four health care sites. The surveys were distributed to nurses who attended EqualHealth’s annual continuing education conference in Port Au Prince, Haiti in November 2014.

The focus groups and the surveys explored Haitian nurses’ attitudes and expectations regarding continuing nursing education in Haiti. The surveys were created in English, translated into French, and back translated to confirm accuracy. Nursing leaders from Haiti were utilized as content experts during the translation process. The anonymous survey contained demographic information that included: age, education (Auxiliary nurse or RN), institution (private, public or NGO) of practice, source of educational/clinical resources, and interest in Continuing Education (CE). Multiple-choice and Likert scale questions were anonymous and completed in private. Questions were designed to gather data on the nurses’ access to current education and resources, and attitudes and beliefs related to CE.

The focus groups and administration of surveys were conducted at the four hospitals. These visits were pre-arranged with nursing leadership at each site. Participation was voluntary and anonymous, and written informed consent was obtained. This sample was based on feasibility and convenience for nurses, size of hospital, and the time of the day for the focus group. Quantitative surveys were completed prior to the focus groups. The focus groups were guided by ten open-ended questions used to generate discussion among participants. The focus groups lasted on average one hour and were led by French and Haitian Creole speaking members of this research team (KL, NR, JC). The focus groups were audio recorded, transcribed, translated into English and analyzed using content analysis. The surveys were analyzed using SPSS 12.0.

## Results

The research participants were diverse in experience, age, and nursing role. The participants ranged from newly-graduated registered nurses with limited experience to more senior staff who had held their nursing positions for many years (Table 1). The participants included nurses in staff roles as well as in leadership and education positions. The sample participants were both registered nurses (n = 72) and nurse auxiliaries (n = 28). Sixty-three percent reported having attended a private nursing school while 37% were graduates of public nursing schools. The majority of participants (51%) reported graduation from three-year nursing programs.

**Table 1:** Demographics of Nurses Surveyed (N = 100).

Demographics	Study Population %

*Job Title*	
Nurse	72%
Auxiliary	28%
*Secondary Education*	
Private	63%
Public	37%
Type of program completed * 5% percent missing	
Two-year nursing program	17.9%
Three-year nursing program	53.7%
Four-year Baccalaureate	13.7%
Other	14.7%
*Year of experience in field*	
0–2 years	27%
3–5 years	36%
6–10 years	23%
More than 10 years	14%
Work Institution * 1% percent missing	
Public Hospital	42.7%
Private Hospital	17.7%
University Hospital	20.8%
NGO	16.7%
Other	2.1%

## Access to Resources

Access to continuing nursing education, for this study, encompassed any post-graduation training including nursing lectures, informal trainings, mentorship, and conferences. It also included self-learning that is attained through accessing nursing books, articles, or online resources. Lack of access to resources was identified as a barrier to continuing education for many of the participants in both the surveys and the focus groups and there were no significant differences between the responses of the RNs and the auxiliaries. One participant noted that participation in continuing education activity did not necessarily guarantee access to the material or resources.

Analysis from our surveys indicated that 72% of nurses lacked a medical library in their workplace, although 48% stated they had nursing books available to them, 38% reported that they used a book for clinical practice at least once per week, 44% stated that they had access to a computer at work, and 40% reported using computers for continuing nursing education needs. Over half of the nurses (65%) reported having access to the internet on their personal phones and reported using their phones to access clinical information. The nurses reported interest in having more access to computers, internet and up-to-date resources in their work environment. When asked about their preferred mode of accessing continuing education, the nurses overwhelmingly reported computer and internet as the most favorable option for learning, followed by lectures and books (Table 2).

**Table 2:** Access to Continuing Educational and Resources.

	Yes %	No%

Do you have a medical library where you work?	29.2%	70.8%
Do you have medical nursing books where you work?	49.5%	50.5%
Is there a computer available for you to use where you work?	50.6%	49.4%
Do you ever use a computer for continuing education at work?	83.3%	16.7%
Do you ever access a computer outside of work for continuing education?	53.8%	46.2%
Do you have access to internet through your phone?	69.9%	30.1%
If yes would you use internet on your phone to access CE resources? (N = 65)	87.1%	12.9%
In the last year have you received any continuing education?	45.8%	54.2%
Do you have a nurse educator where you work?	62.1%	37.9%
Does your nurse educator provide you with clinical lecture/updates?	88.2%	11.8%
Does your hospital provide you time off to attend nursing conferences?	36.9%	63.1%
Does your hospital provide you with financial support to attend conferences?	16%	84%
Would you be interested in having more continuing education available to you?	96.8%	3.2%

## Interest in Continuing Education

The survey results regarding interest in continuing education revealed that 97% of the participants were interested in access to continuing education opportunities. This interest was echoed in the focus groups with one participant stating, “Continuing education should not be merely seen as a quality improvement. We should encourage and provide opportunities for all nursing staff to engage in certain number of continuing education credits per year to keep working. Better quality of care would have a beneficial effect on the Haitian health system.” Comments from other participants were similar, stating that, “continuing education and training should be the most important thing,” and “for me it’s non-negotiable but essential.” These opinions were reaffirmed by the participants in all the focus groups.

## Barriers to Education

Multiple barriers to continuing education were identified and include work schedule, family obligations, time, lack of institutional support, cost, and lack of funding from the institutions. These findings are consistent with the literature. While 68% of the nurses surveyed reported having access to a clinical nurse educator at their institutions, they felt that they could benefit from more education time. Thirty-three percent of participants reported receiving monthly clinical updates from their nurse educators; yet, they reported a strong interest in more specialized trainings that corresponded to their work areas (pediatric, emergency medicine, and cardiology for example). Lack of financial support from their institutions to attend conferences was reported as a barrier by the participants. This was reported as one of the greatest barriers to continuing education. Lack of time to attend conferences either on-site or outside of their institution was noted as another barrier. Heavy workloads and geographical distance of the continuing education programs are factors that negatively impacted their ability to attend. Nurses also reported that their environment was not always conducive to learning; however, they reported increased positive relationships with other staff and collaboration across disciplines.

## Lack of a Standardized Nursing Education System

Standardization of nursing education by the Ministry of Health (MOH) in Haiti was an area that was discussed widely during the focus groups. At the time of this study, the MOH was in the process of creating a more standardized four-year curriculum for nurses. Many nurses reported lack of knowledge about these changes and wanted increased communication from the MOH so that they could be better informed. However, they were excited to hear about the change and felt these changes would be positive for the country. The participants believed having more standardized education with a stronger curriculum would improve nursing training and weed out poor-quality schools.

Many of the participants expressed concern over the state of some of the nursing schools in Haiti. They felt that there were too many nursing schools and the goal of some of them was not to educate but to primarily generate revenue. Although there are over four hundred nursing schools in Haiti, only 35 schools including the four state schools are recognized by the Ministry of Health [19]. One nurse stated, “The quality of the training is diminishing considerably because there are some people giving this training who are not even in the field of nursing, so with nursing schools popping up left and right, multiplying, any mercenary can have a nursing school.” Another nurse expressed similar feelings and concerns, “the schools that these kinds of people operate are not looking for quality, but quantity [of students] it’s a business.” Nurses also reported a burden on them to “educate” the nurses that were taught in schools with questionable curriculum. This sentiment resonated in all four focus groups. Many expressed strong feelings that the profession was being devalued and was no longer one of respect. Many went one step further and associated lack of respect and mistrust from physician colleagues as the result of the problem existing in the nursing education system, mainly the schools. The nurses also compared the differences in the educational system with regards to medical and nursing education, “There is one state and three private medical schools. There are more than 400 private nursing schools operating in Haiti.”

## Nurse-Physician Collaboration

Nurse-physician collaboration has been noted in the literature as a tool to improve patient outcomes [25]. The subject of collaboration came up in the focus groups; it centered on the relationships that exist between doctors and nurses and how it affects patient care and the healthcare setting overall. There were stories of positive collaboration and teamwork among disciplines, and all agreed that positive collaboration lead to better patient outcomes and increased job satisfaction. “Good sharing of ideas contributes to patient improvement,” stated one participant. Another nurse discussed the benefits of team rounds among disciplines, “When we round, we share our own personal knowledge, our knowledge about the case, and if there was something that you forgot or didn’t realize, you can then learn it or realize it during rounds.” The participants also reported stories of non-effective communication where open discussions and collaboration were not engaged in and how it negatively affected the staff and environment. Participants felt that if self-advocacy and mentoring was taught in schools that could have a positive influence on physician-nursing interactions.

## Continuing Education

Many of the participants voiced concern that physicians received far more support from their institutions than the nurses in regards to continuing education. Although they did not provide any data to support this statement, they believed that the medical leadership encourages their staff physicians to attend continuing education while not giving that same opportunity to the nurses. One participant stated, “For the MDs it seems so much easier, but for the nurses it is much harder.” Yet, the participants acknowledged the importance of continuing education while highlighting the many barriers that they faced including the balance between attending a conference with staffing their institution. One stated, “There must be a time for training and a time for working because both are very important.” Overall nurses acknowledged needing more support from their institutions and leadership to attend CE conferences.

## Discussion

The literature review conducted at the beginning of this project did not reveal any other research that looked at Haiti and continuing education for nurses. However, since that time Clark et al., (2015) published an article about their work in Haiti that highlighted the need for increased continuing education for nurses through a case study. Many studies have shown that access to continuing nursing education increases job satisfaction and decreases nursing turnover [26,27,28]. The main goal of CE is to improve nursing practice and patient care [29]. Many studies have proven that CE courses have a positive effect on nursing knowledge and patient care outcome [30]; Haitian nurses believe that, too. They also feel that standardized education and access to continuing education will lead to better patient care and increased job satisfaction in their communities.

This research revealed that the Haitian nurses are interested in continuing education and highlighted the many barriers that these nurses faced. These barriers included lack of resources, lack of staff, strained physician-nurse relationships, high workloads, heavy patient assignments, lack of time, and lack of support from their leaders and institutions.

Ensuring there are more opportunities for CE with practice guidelines disseminated by the Ministry of Health offers the opportunity to advance CE efforts. A more standardized basic education across the country would help improve the quality of healthcare throughout the country. One Haitian nurse leader, Roodeline Valcourt, expressed her thoughts about the changes to curriculum on a popular website: Global Health Delivery. She stated, “This new curriculum is the realization of a common dream. This is a new bend in the road for training nurses in Haiti. Establishing a bachelor’s degree four-year program in nursing is an essential component in elevating the collective standard of professional practice [31].”

## Conclusion

Our physician colleagues from EqualHealth have found similar and synergistic goals for advancing medical education in Haiti [31]. Clark et al., (2015) suggests a need for policy-makers in settings that are resource-limited proactively invest in nursing continuing education. Haitian nurses are talented, intelligent, and dedicated. We at EqualHealth have been inspired by their commitment to their patients and to a healthier Haiti. Haitian nurses value, and are committed to lifelong learning. CE is not only good for the nursing profession and the institutions where nurses practice, but is proven to improve patient outcomes [4,5,7]. It is well known that continuing education is needed in all aspect of nursing; it is vital in a country such as Haiti in which there are communities in which nurses are sole health-care providers.
